# Case Report: When muscle edema is not a myositis flare: *Legionella* pneumonia complicated by rhabdomyolysis and acute kidney injury in long-standing polymyositis

**DOI:** 10.3389/fmed.2026.1952498

**Published:** 2026-10-05

**Authors:** Jing Zhang, Lina Zhang, Sheng-Guang Li, Yadan Zou, Ting Long, Ruohan Yu, Yanfeng Zhang, Ji Li

**Affiliations:** Department of Rheumatology and Immunology, Peking University International Hospital, Beijing, China

**Keywords:** kidney injury, case report, *Legionella pneumophila*, muscle MRI, polymyositis, rhabdomyolysis, targeted next-generation sequencing

## Abstract

*Legionella pneumophila* is an important cause of severe community-acquired pneumonia and can produce extrapulmonary manifestations that obscure the primary diagnosis. Diagnostic error is especially likely in patients with established idiopathic inflammatory myopathy, because fever, myalgia, weakness, creatine kinase elevation and muscle edema may be attributed to autoimmune flare. We report a 70-year-old woman with a 25-year history of polymyositis receiving long-term corticosteroids and azathioprine who developed high fever, severe bilateral thigh pain and progressive proximal weakness after recent travel. On transfer, she had anuria with bladder distension, dark urine, diffuse posterior-thigh muscle edema, pneumonia, hyponatremia, metabolic acidosis, myoglobinemia and acute kidney injury. *Legionella* pneumonia with rhabdomyolysis and multifactorial acute kidney injury was suspected clinically, and Legionella-active antimicrobial therapy, hydration, urinary drainage and urine alkalinization were started before microbiological confirmation. A targeted sequencing-based respiratory pathogen assay on sputum collected on Day +4 detected *L. pneumophila* (145 sequences; estimated concentration <10^3 copies/mL) and respiratory syncytial virus A (266,036 sequences; 10^5 copies/mL), raising the possibility of viral-bacterial coinfection. *Legionella* urinary antigen, targeted PCR and culture were not documented. After infection and renal injury stabilized, methylprednisolone and intravenous immunoglobulin were used because residual inflammatory myopathy activity remained in the differential. However, creatine kinase and myoglobin had already declined, so subsequent improvement cannot be attributed specifically to immunomodulation. Creatinine fell from 538 μmol/L on Day 0 to 110 μmol/L on Day +9 and 78 μmol/L on Day +30; follow-up chest CT on Day +14 showed marked improvement. This case shows that muscle MRI edema is contextual rather than etiologic. In immunosuppressed myositis patients, pneumonia plus hyponatremia, myoglobinuria and renal injury should prompt early Legionella-active treatment while autoimmune activity is reassessed after infection control.

## Introduction

Legionnaires’ disease remains a clinically important form of community-acquired pneumonia because early symptoms are often non-specific and standard beta-lactam regimens do not cover intracellular *Legionella* species. Contemporary pneumonia guidelines emphasize empiric coverage for atypical pathogens when severity, epidemiologic setting or clinical pattern warrants it, and recent guidance continues to frame diagnostic testing as a complement to timely treatment rather than a prerequisite for treatment initiation (1, 2). Classic clues include high fever, gastrointestinal or neurologic symptoms, hyponatremia, liver enzyme elevation, high inflammatory markers and limited response to beta-lactams, but none is individually diagnostic (3–7).

Rhabdomyolysis with acute kidney injury (AKI) is an uncommon but consequential extrapulmonary manifestation of *Legionella* infection. The triad of pneumonia, rhabdomyolysis and renal failure has been repeatedly reported and may be fatal when recognition is delayed (8–11). The diagnostic problem is amplified in rheumatology practice. Patients with idiopathic inflammatory myopathies may already have proximal weakness, elevated muscle enzymes and magnetic resonance imaging (MRI) muscle edema; chronic corticosteroids or immunosuppressants further raise concern for severe infection (12–14). We report a patient with long-standing polymyositis in whom *Legionella* pneumonia produced rhabdomyolysis, AKI and diffuse muscle edema that could easily be mistaken for a myositis flare. The case is reported in accordance with CARE guidance (15, 16).

## Case description

A 70-year-old woman had been diagnosed with polymyositis 25 years earlier after myalgia, weakness, creatine kinase (CK) elevation and myopathic electromyography; no muscle biopsy had been performed. At the onset of this episode, she was taking azathioprine 50 mg/day and one-half tablet of an oral corticosteroid daily. The corticosteroid tablet strength, pre-episode CK level and formal baseline manual muscle testing score were not documented. Approximately one month after travel, she developed 8 days of high fever up to 41°C, severe bilateral thigh pain and progressive proximal lower-limb weakness, with difficulty lifting and bending the legs. Detailed travel-exposure information, including hotel accommodation, aerosolized-water exposure and illness among travel companions, was unavailable.

Five days before transfer, she attended an outside hospital. CRP was >200 mg/L, procalcitonin was 10.6 ng/mL and CK was 865 U/L. On Day -4, serial records documented CRP 248 mg/L, procalcitonin 10.5 ng/mL, CK 856 U/L, serum myoglobin >900 ng/mL, sodium 126.9 mmol/L and creatinine 91 μmol/L. Initial chest radiography or CT was not documented. The illness was interpreted as polymyositis relapse with infection, and intravenous ertapenem was started. Fever resolved within 4 days, but muscle pain and weakness did not improve. She became unable to turn over or walk and had complete anuria for 2 days before transfer to our hospital.

At transfer, vital signs were stable. There was no superficial lymphadenopathy. Chest auscultation revealed coarse breath sounds with prominent wet crackles. The lower abdomen was distended over the bladder, without tenderness. Muscle tone was normal, but the limbs were diffusely tender; proximal muscle strength was Medical Research Council grade 3/5. Deep tendon reflex grades were not documented, and pathological reflexes were absent. Catheterization drained approximately 2,000 mL of dark tea-colored urine. Urine myoglobin was reported as markedly elevated, supporting myoglobinuria, but the numerical value and laboratory reference interval were unavailable.

Laboratory evaluation showed leukocytosis of 12.74 × 10^9/L with 93.5% neutrophils, erythrocyte sedimentation rate 95 mm/h and conventional CRP 147.81 mg/L; a high-sensitivity CRP result of 180.2 mg/L was also recorded. Sodium was 125 mmol/L, chloride 91 mmol/L, bicarbonate 15.9 mmol/L, calcium 1.83 mmol/L, phosphate 2.73 mmol/L and magnesium 1.18 mmol/L. Creatinine was 538 μmol/L, urea 40.71 mmol/L and uric acid 739 μmol/L. CK was 1,809 U/L, lactate dehydrogenase 546 U/L, serum myoglobin 780.4 ng/mL, CK-MB 11.9 ng/mL, high-sensitivity troponin T 77.4 ng/L and N-terminal pro-B-type natriuretic peptide 423.5 pg/mL. Fibrinogen was 704 mg/dL, fibrin degradation products were 28.1 μg/mL and D-dimer was 3,336 ng/mL. SARS-CoV-2 and influenza antigen tests were negative. Myositis serology showed anti-Jo-1 positivity at 439.65 U/mL (reference <185 U/mL), together with anti-U1-RNP, anti-centromere, anti-SSA/Ro60 and anti-SSA/Ro52 positivity. Antinuclear antibody was positive at 1:640 with a granular pattern and 1:160 with a cytoplasmic granular pattern; rheumatoid factor, anti-cyclic citrullinated peptide antibody and antineutrophil cytoplasmic antibody were normal or negative. Anti-Jo-1 positivity with chronic interstitial lung disease was compatible with an antisynthetase-spectrum or overlap-myositis phenotype, although the historical diagnosis of polymyositis remained the clinical anchor (12). Elevated high-sensitivity troponin T and a small pericardial effusion raised the possibility of cardiac involvement, but electrocardiography, troponin I, echocardiography and cardiac MRI findings were not documented, and myocarditis was not established.

Bilateral thigh MRI demonstrated diffuse posterior-compartment muscle edema (Figures 1A, B). Chest CT showed chronic interstitial fibrotic changes, left upper-lobe and right lower-lobe interstitial pneumonia with patchy consolidation, mild bronchiectasis and a small pericardial effusion (Figure 1C). Selected longitudinal laboratory trends are shown with relative-day labels in Figure 1E, and the treatment sequence relative to laboratory improvement is summarized in Figure 2 and Table 1.

**Figure 1:**
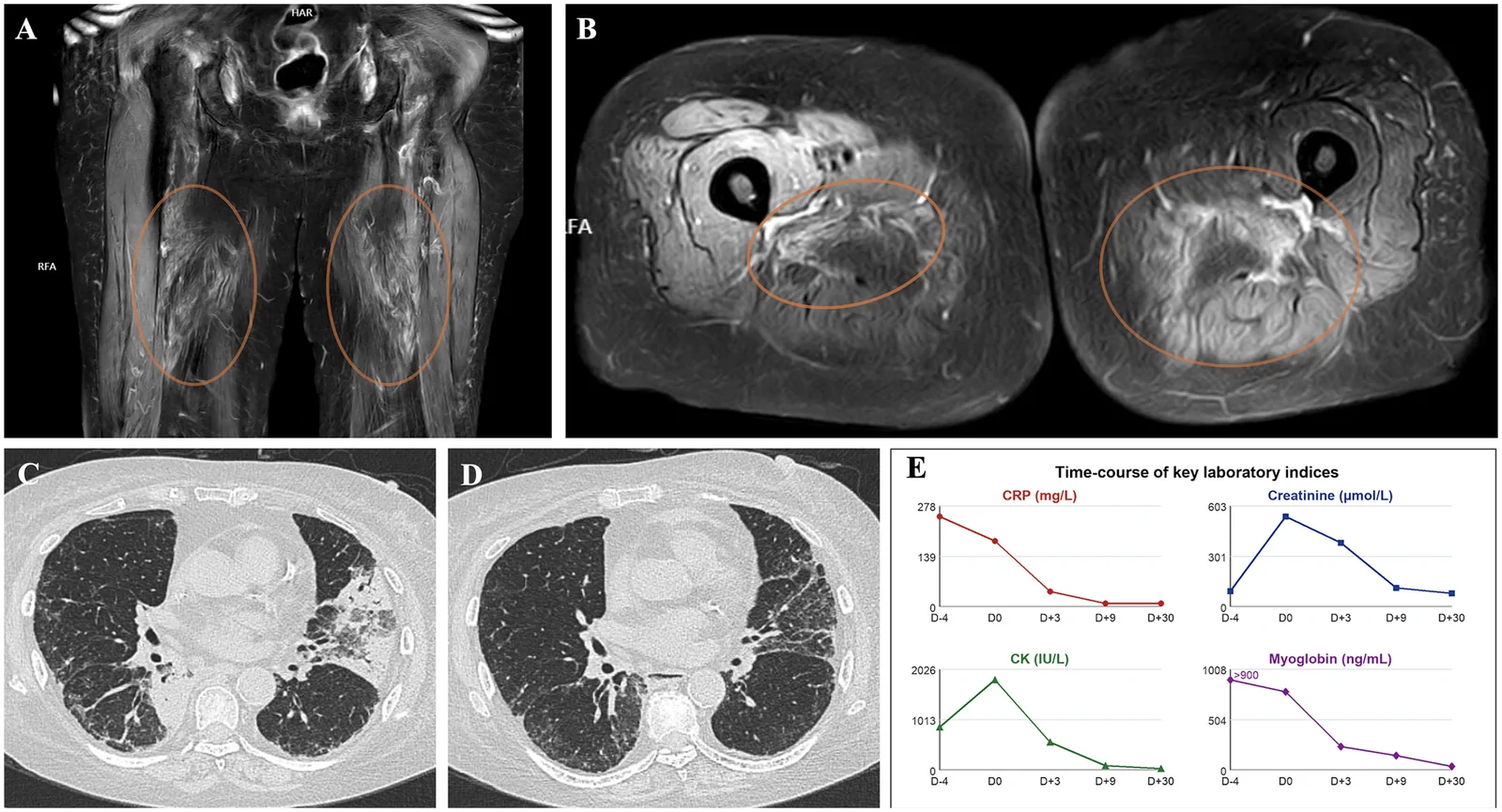
Muscle and pulmonary imaging with laboratory response in *Legionella* pneumonia complicated by rhabdomyolysis and acute kidney injury. **(A,B)** Bilateral thigh MRI demonstrates diffuse posterior-compartment and multi-compartment muscle edema outlined in orange-red, consistent with extensive muscle injury but not specific for autoimmune flare. **(C)** Chest CT at transfer shows patchy consolidation and interstitial inflammatory changes. **(D)** Follow-up chest CT on Day +14 shows marked radiographic improvement. **(E)** Four small-multiple plots show CRP, creatinine, CK and serum myoglobin on Days −4, 0, +3, +9 and +30. Muscle and renal indices were already improving during antimicrobial and supportive therapy before later immunomodulation shown in Figure 2. The first serum myoglobin value exceeded 900 ng/mL. Alt text: Five-panel composite showing orange-red outlines around areas of bilateral thigh muscle edema on MRI, paired chest CT images before and after treatment, and four relative-day laboratory trajectories demonstrating improvement in inflammation, muscle injury and renal function.

**Figure 2:**
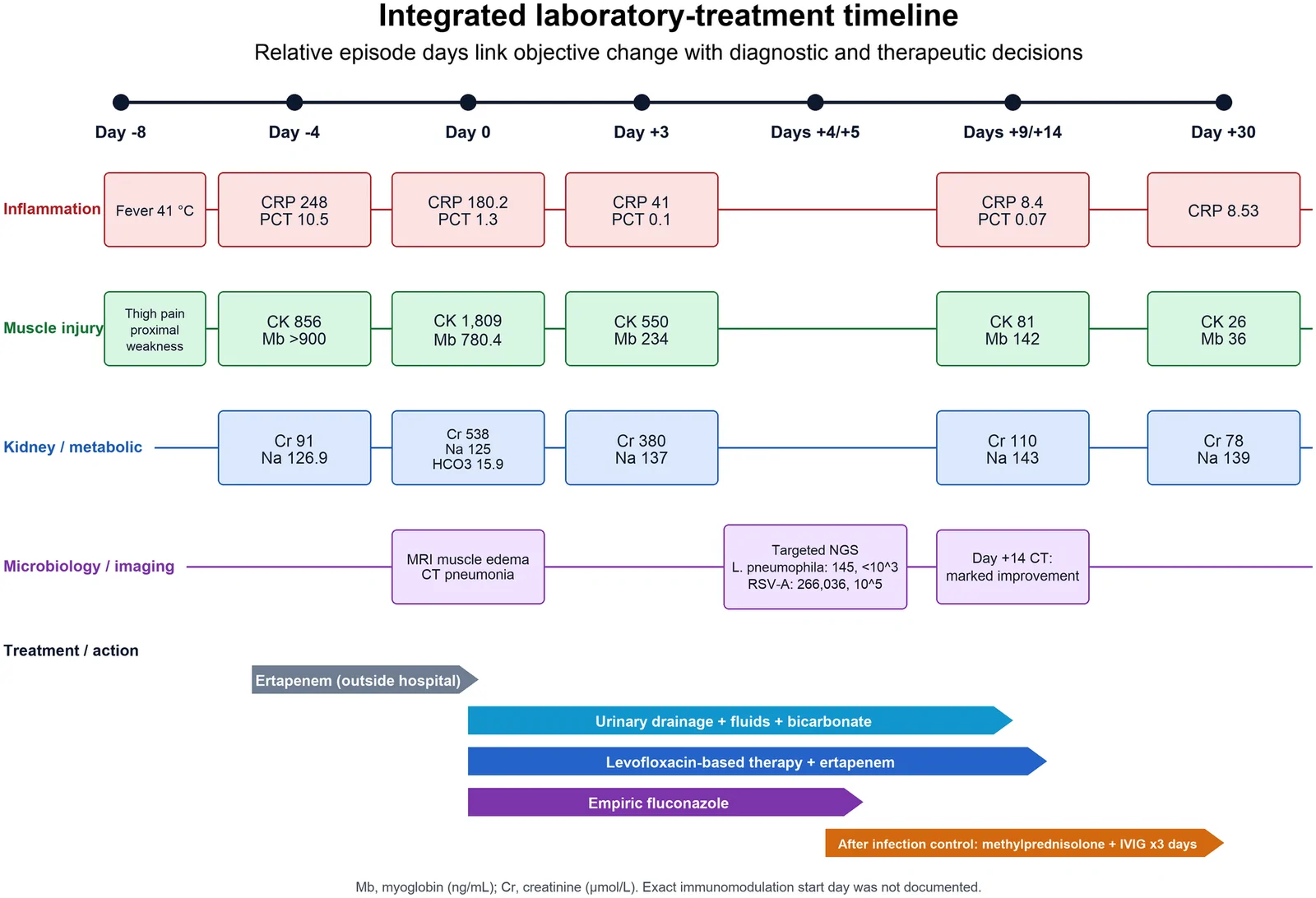
De-identified relative timeline of diagnostic pivot and treatment sequence. The timeline links symptoms and serial inflammatory, muscle, renal and metabolic indices with urinary drainage, fluids, bicarbonate, antimicrobial therapy, targeted respiratory sequencing and later immunomodulation. Sputum was collected on Day +4 and reported on Day +5; the assay detected *L. pneumophila* together with a substantially stronger RSV-A signal. Chest CT showed marked improvement on Day +14, and laboratory recovery continued through Day +30. Alt text: Relative-day timeline showing objective laboratory changes, Legionella-active treatment before the targeted sequencing report, possible RSV-A coinfection, delayed methylprednisolone/intravenous immunoglobulin after infection control and recovery without dialysis.

**Table 1:** CARE-style timeline of the episode of care.

Relative day	Clinical and laboratory findings	Diagnostic interpretation	Management and outcome
Day -8	High fever up to 41 °C after recent travel, with severe bilateral thigh myalgia and progressive proximal weakness. Specific travel accommodation, aerosolized-water exposure and companion-illness information were not documented.	Severe systemic inflammation in an immunosuppressed patient; infection had to remain high in the differential despite known polymyositis.	Initial presentation before transfer.
Days -5 to -4	At the outside hospital, CRP was >200 mg/L, procalcitonin 10.6 ng/mL and CK 865 U/L. Day -4 values were CRP 248 mg/L, procalcitonin 10.5 ng/mL, CK 856 U/L, serum myoglobin >900 ng/mL, creatinine 91 μmol/L and sodium 126.9 mmol/L; initial pulmonary imaging was not documented.	Severe inflammation with early muscle injury and hyponatremia supported infection-associated muscle injury or infection-triggered autoimmune activity.	Intravenous ertapenem was started for presumed infection with possible myositis relapse.
Days -4 to -1	Fever resolved within 4 days, but thigh pain and weakness progressed; the patient became bedbound and had complete anuria for 2 days.	Defervescence did not equal disease control. Progressive myoglobinuric injury and urinary retention/anuria required urgent reassessment.	Transferred for further evaluation.
Day 0	Proximal strength MRC grade 3/5, diffuse muscle tenderness, coarse crackles, bladder distension. Catheterization drained 2,000 mL of dark tea-colored urine; urine myoglobin was reported as markedly elevated, but the numerical urine value was unavailable.	Dark urine plus muscle pain and acute kidney injury supported rhabdomyolysis rather than uncomplicated myositis relapse.	Immediate urinary drainage and supportive management.
Day 0 labs	CK 1,809 U/L; serum myoglobin 780.4 ng/mL; creatinine 538 μmol/L; urea 40.71 mmol/L; sodium 125 mmol/L; bicarbonate 15.9 mmol/L; conventional CRP 147.81 mg/L and high-sensitivity CRP 180.2 mg/L.	Hyponatremia, pneumonia, severe inflammation, myoglobinuria and renal injury formed a pattern compatible with *Legionella* pneumonia complicated by rhabdomyolysis-associated AKI.	Bilateral thigh MRI and chest CT were obtained.
Day 0 imaging	Thigh MRI showed diffuse posterior-compartment muscle edema; chest CT showed interstitial pneumonia with patchy consolidation and mild bronchiectasis.	MRI edema indicated muscle injury but was not specific for autoimmune flare; concurrent pneumonia shifted the diagnostic priority toward infection.	Empiric Legionella-active therapy was started rather than waiting for pathogen confirmation.
Day +3	CRP 41 mg/L, procalcitonin 0.1 ng/mL, CK 550 U/L, serum myoglobin 234 ng/mL, creatinine 380 μmol/L and sodium 137 mmol/L.	Inflammatory, muscle and renal indices were already improving during antimicrobial and supportive treatment.	Levofloxacin-based therapy, urinary drainage, fluids and bicarbonate continued; no dialysis was required.
Days +4 to +5	A targeted sequencing-based respiratory assay detected *L. pneumophila* (145 sequences; <10^3 copies/mL) and RSV-A (266,036 sequences; 10^5 copies/mL). EBV, CMV and three Candida species were categorized as conditional pathogens.	The Legionella result supported the pretest diagnosis, while the stronger RSV-A signal indicated possible viral-bacterial coinfection. Respiratory Candida detection did not establish invasive fungal disease.	Legionella-active therapy had already begun. Fluconazole was empirical; methylprednisolone and IVIG were introduced only after infection and renal injury stabilized.
Days +9 to +30	By Day +9, CRP was 8.4 mg/L, CK 81 U/L, serum myoglobin 142 ng/mL and creatinine 110 μmol/L. Chest CT on Day +14 showed marked improvement. By Day +30, CK was 26 U/L, myoglobin 36 ng/mL and creatinine 78 μmol/L.	Sustained biochemical and radiographic recovery supported reversible infection-associated systemic injury; the contribution of active autoimmune myositis remained uncertain.	Strength improved clinically. No renal replacement therapy or serious treatment-related adverse event occurred.

## Diagnostic assessment

The initial diagnostic question was whether the patient had inflammatory myopathy activity, infection-induced rhabdomyolysis, or both. The historical inflammatory myopathy diagnosis, anti-Jo-1 positivity, chronic interstitial lung disease, proximal weakness, CK elevation and diffuse MRI muscle edema kept autoimmune activity in the differential. Anti-Jo-1 positivity characterized the underlying phenotype but did not establish an acute flare. CK and myoglobin were already declining with antimicrobial and supportive treatment before immunomodulation, and repeat MRI, electromyography, aldolase testing or muscle biopsy was not performed. Several features were difficult to reconcile with flare alone: fever to 41°C, very high CRP and procalcitonin, pneumonia on CT, hyponatremia, dark urine, myoglobinemia, AKI and progression despite defervescence on a beta-lactam regimen. MRI muscle edema was therefore interpreted as evidence of muscle injury but not proof of autoimmune myositis. Skeletal muscle hyperintensity on fluid-sensitive sequences occurs in inflammatory, infectious, ischemic, toxic, traumatic and metabolic muscle injury (14, 17–19).

*Legionella* pneumonia complicated by rhabdomyolysis-associated AKI was suspected because the pulmonary syndrome, hyponatremia, severe systemic inflammation, limited beta-lactam activity and myoglobinuric renal injury formed a coherent pattern (8, 20, 21). *Legionella* urinary antigen testing, targeted PCR/nucleic acid amplification testing and conventional culture were not documented. The patient was initially too weak to produce sputum, and treatment could not wait. Sputum collected on Day +4 was analyzed with a targeted sequencing-based respiratory pathogen assay covering a predefined pathogen panel; the Day +5 report detected *L. pneumophila* (145 sequences; estimated concentration <10^3 copies/mL) and respiratory syncytial virus A (RSV-A; 266,036 sequences; 10^5 copies/mL) as pathogenic organisms. The assay also categorized Epstein–Barr virus, cytomegalovirus and three Candida species as conditional pathogens and detected no resistance or virulence genes. Because RSV-A produced a substantially stronger signal, viral-bacterial coinfection remained possible. The *L. pneumophila* result was interpreted as supportive rather than exclusive evidence, in conjunction with the clinical syndrome and response to levofloxacin. Relative abundance, genome coverage and the laboratory positivity threshold were not provided. Sequencing-based results in severe pneumonia should be interpreted with clinical context rather than used in isolation (22). Diagnostic alternatives and the reasoning process are shown in Table 2.

**Table 2:** Diagnostic reasoning and literature context for legionella-associated rhabdomyolysis in a patient with polymyositis.

Evidence theme	Representative literature	Relevance to this case
Recognition of the triad	*Legionella* pneumonia, rhabdomyolysis and AKI have been repeatedly described as an uncommon but high-risk constellation (8, 9, 11).	The present case adds a myositis-specific diagnostic trap: the muscle symptoms and MRI edema were initially plausible as autoimmune flare.
Severity spectrum	Reports range from preserved renal function despite extreme CK elevation to severe AKI requiring continuous renal replacement therapy (27, 29).	Early fluids, urine alkalinization and Legionella-active antimicrobials coincided with renal recovery without renal replacement therapy.
Sequencing-supported diagnosis	Published reports used metagenomic sequencing to identify *Legionella* when routine methods were delayed or limited (20–22); sequencing-supported Legionella-rhabdomyolysis-AKI cases have already been described.	The present targeted assay detected *L. pneumophila* together with a much stronger RSV-A signal. It supported the clinical diagnosis but did not establish exclusive *Legionella* infection; novelty lies in the inflammatory-myopathy diagnostic trap.
Diagnostic clues	Hyponatremia, high inflammatory markers, pneumonia, extrapulmonary findings and limited response to beta-lactams are repeatedly discussed as *Legionella* clues (3, 5–7).	The same clues were present before the targeted sequencing report and are actionable in immunosuppressed rheumatology patients.
Muscle MRI interpretation	Muscle edema is useful for localizing muscle injury in idiopathic inflammatory myopathies but is not disease-specific (14, 17, 18).	The case argues for reading MRI edema through the whole clinical syndrome rather than treating it as proof of flare.

## Therapeutic intervention, follow-up and outcomes

Management prioritized urinary drainage, rhabdomyolysis-associated renal injury and untreated atypical pneumonia. The cause of urinary retention remained undetermined. Renal ultrasonography reportedly showed no obstruction, but detailed post-catheter urine-output data were unavailable. Urine output improved and serum creatinine decreased after catheterization together with intravenous fluids, bicarbonate for urine alkalinization and empiric antimicrobial therapy including levofloxacin and ertapenem. Because these interventions occurred together, the relative contributions of transient retention and rhabdomyolysis-associated tubular injury could not be quantified. Fluconazole was given empirically in the setting of chronic immunosuppression and severe pneumonia. The subsequent respiratory assay categorized Candida tropicalis, Candida albicans and Nakaseomyces glabratus as conditional pathogens, but respiratory detection alone did not establish invasive fungal infection, and no candidemia or other invasive candidiasis was documented. Rhabdomyolysis-associated AKI reflects myoglobin-mediated tubular injury, renal vasoconstriction and intratubular cast formation; early volume expansion remains central to prevention and recovery (23).

After infection control and renal stabilization, the team reassessed residual weakness in the context of long-standing inflammatory myopathy. Methylprednisolone 40 mg daily and intravenous immunoglobulin 20 g/day for 3 days (total 60 g) were administered for possible residual inflammatory activity rather than a proven flare; body weight was not documented, precluding expression of the dose in g/kg. CK, serum myoglobin and creatinine had already fallen to 550 U/L, 234 ng/mL and 380 μmol/L by Day +3, before immunomodulation. They declined further to 81 U/L, 142 ng/mL and 110 μmol/L by Day +9 and to 26 U/L, 36 ng/mL and 78 μmol/L by Day +30. CRP fell from 180.2 mg/L on Day 0 to 41 mg/L on Day +3 and 8.4 mg/L on Day +9; sodium normalized by Day +3. Follow-up chest CT on Day +14 showed marked absorption of the pulmonary lesions. Muscle strength improved clinically, although no formal follow-up MMT score was obtained. No renal replacement therapy was required, and no serious treatment-related adverse event occurred. Because biochemical improvement preceded immunomodulation and no repeat MRI, electromyography, aldolase test or muscle biopsy was performed, recovery cannot be attributed specifically to methylprednisolone and intravenous immunoglobulin.

## Discussion

This case has three practical messages. First, *Legionella* should be considered when pneumonia and rhabdomyolysis occur together, especially when hyponatremia, very high inflammatory markers, myoglobinuria and limited beta-lactam activity are present. The literature contains fatal and reversible examples of Legionella-associated rhabdomyolysis and AKI, including sequencing-supported diagnoses (9–11, 20, 21, 24–29). The distinctive feature here is the diagnostic confusion created by established inflammatory myopathy, anti-Jo-1 positivity, chronic interstitial lung disease and diffuse MRI muscle edema. The concurrent RSV-A signal prevents the respiratory assay from being interpreted as evidence of *Legionella* infection in isolation, but it does not negate the Legionella-compatible clinical bundle or the response to Legionella-active therapy.

Second, MRI muscle edema should be read as a sensitive map of muscle injury, not as a stand-alone etiologic diagnosis. In idiopathic inflammatory myopathies, MRI can identify active muscle inflammation, guide biopsy and help monitor disease activity; however, edema is also seen in rhabdomyolysis, denervation, trauma, infection, ischemia and metabolic injury (14, 17, 18). In this patient, the distribution of edema and the history of polymyositis made flare plausible, but the simultaneous pulmonary infection, hyponatremia, dark urine and AKI made infection-driven rhabdomyolysis more urgent. Persistent weakness alone was insufficient to prove active myositis, because clinical recovery of strength may lag behind biochemical improvement after rhabdomyolysis and critical illness. A reflex escalation of immunosuppression before controlling infection could have been hazardous. The sequence used here–treat infection and rhabdomyolysis first, then reassess inflammatory activity–offers a pragmatic approach for similar rheumatology cases.

Third, the sequencing result was supportive rather than the therapeutic trigger. Unlike the unbiased metagenomic sequencing used in some published *Legionella* cases (20–22), the assay in this patient targeted a predefined respiratory pathogen panel. It provided sequence counts and estimated concentrations but not relative abundance, genome coverage or a documented positivity threshold. *L. pneumophila* was detected at 145 sequences and <10^3 copies/mL, whereas RSV-A was detected at 266,036 sequences and 10^5 copies/mL. These findings support possible viral-bacterial coinfection and require clinical interpretation rather than attribution of the entire pulmonary syndrome to a single assay result. Legionella-active treatment had already begun because sputum was initially unobtainable and the clinical pattern was compelling. The later report supported continuation of therapy and reduced anchoring on myositis flare. Detection of EBV, CMV and Candida species in a respiratory sample did not by itself establish active systemic viral or invasive fungal disease.

A practical way to frame the case is to separate three questions that are often compressed at the bedside. The first question is whether there is active muscle injury; in this patient the answer was clearly yes, based on pain, weakness, CK elevation, serum and urine myoglobin and MRI edema. The second question is whether the muscle injury is autoimmune, infectious, toxic, ischemic or multifactorial; the answer cannot be supplied by MRI alone. The third question is which process will harm the patient fastest if undertreated. At transfer, the immediately dangerous process was not a theoretical under-treatment of polymyositis but established rhabdomyolysis with AKI in the setting of pneumonia. This priority order justified fluids, urinary drainage, alkalinization and Legionella-active antimicrobial therapy before escalation of immunosuppression.

The case also highlights a subtle but important difference between a disease flare and a disease-context complication. A patient with polymyositis can certainly flare during or after infection, and infection itself can amplify inflammatory muscle symptoms. However, labeling the whole syndrome as flare risks missing a treatable pathogen. In the present case, several data points acted as a ‘Legionella-compatible bundle': severe pneumonia, hyponatremia, very high inflammatory markers, limited beta-lactam activity, myoglobinuria and renal injury. Individually, each finding is non-specific; together, they created a pattern strong enough to treat empirically. This pattern-based reasoning is especially relevant in older or immunosuppressed patients, in whom typical pneumonia symptoms may be incomplete and extrapulmonary findings may dominate the consultation.

The mechanism of Legionella-associated rhabdomyolysis is not fully established. Proposed explanations include direct bacterial or toxin-mediated muscle injury, cytokine-mediated myocyte damage, microvascular injury, tissue hypoxia and metabolic stress, followed by myoglobin-mediated tubular toxicity and AKI (8, 23). The moderate CK elevation in this patient compared with some published cases does not weaken the diagnosis, because renal injury depends not only on CK magnitude but also on volume status, timing, tubular stress, sepsis physiology, urinary retention and comorbid susceptibility. The prompt improvement after urinary drainage, hydration, alkalinization and Legionella-active therapy supports a reversible systemic injury rather than irreversible intrinsic renal disease.

The renal presentation deserves particular attention. The patient had bladder distension with immediate drainage of approximately 2,000 mL dark urine and biochemical AKI with myoglobinemia. Renal ultrasonography reportedly showed no obstruction, making fixed postrenal obstruction less likely, but the cause of retention and its transient contribution to renal dysfunction remained uncertain. Urinary retention and rhabdomyolysis-related tubular stress may therefore have converged, and AKI is best described as multifactorial. The management implications were aligned: restore urine flow, expand intravascular volume, reduce tubular myoglobin exposure where possible and remove the infectious driver. The rapid biochemical recovery after this bundled approach is a key outcome of the case.

The principal contribution of this case is the rheumatology-facing diagnostic lesson rather than another demonstration of the Legionella-rhabdomyolysis association. Current myositis classification and imaging literature emphasize proximal weakness, enzyme elevation and MRI muscle edema in idiopathic inflammatory myopathy assessment (12, 14, 19). This case shows the boundary of those tools: when pulmonary infection, hyponatremia and AKI coexist, the same muscle findings are non-specific evidence of injury until infection has been addressed. Anti-Jo-1 positivity and chronic interstitial lung disease also illustrate why a patient historically diagnosed with polymyositis may now be viewed within an antisynthetase-spectrum or overlap-myositis framework. The infection-first diagnostic principle is transferable to autoimmune patients receiving corticosteroids, azathioprine or other immunosuppressants.

The main strength of this report is the integrated clinical, imaging, renal and microbiological account of a patient whose autoimmune history created a realistic diagnostic pitfall. It also illustrates a cautious management order: stabilize infection, urinary retention and rhabdomyolysis before considering immunomodulation for possible residual inflammatory myopathy activity. As a single case, it cannot establish causality. *Legionella* urinary antigen, targeted PCR and culture were not obtained or documented, and the targeted sequencing assay did not report relative abundance, genome coverage or a positivity threshold. The strong RSV-A signal leaves viral-bacterial coinfection possible. Formal baseline and follow-up MMT scores, quantitative urine myoglobin, aldolase, repeat electromyography, repeat muscle MRI, muscle biopsy, deep tendon reflex grades, detailed post-catheter urine output and advanced cardiac testing were unavailable. Consequently, the relative contributions of transient urinary retention and myoglobin-mediated tubular injury, as well as the presence of active autoimmune myositis after infection control, cannot be determined precisely. Nevertheless, diffuse muscle edema in an immunosuppressed myositis patient with pneumonia should not be assumed to represent flare.

## Patient perspective

The patient described the sudden inability to walk, dark urine and loss of urine output as frightening because they resembled a severe deterioration of her long-standing muscle disease. She expressed relief when kidney function and strength improved after infection-focused treatment and understood that future episodes of fever with muscle pain should prompt early medical assessment rather than assuming a myositis relapse.

## Conclusion

*Legionella* pneumonia can masquerade as inflammatory myopathy activity when it presents with myalgia, weakness, CK elevation and diffuse muscle edema. In immunosuppressed patients, pneumonia with hyponatremia, myoglobinuria and AKI should prompt early Legionella-active therapy, urinary drainage when retention is present, and rhabdomyolysis management while microbiological testing is pursued. A targeted respiratory sequencing result should be interpreted with its assay design, signal strength and co-detected organisms; in this patient, the strong RSV-A signal was compatible with coinfection rather than exclusive *Legionella* disease. Immunomodulation can be reconsidered after infection control if residual inflammatory activity remains plausible, but treatment response should not be attributed to immunomodulation without objective evidence of active myositis.

## Data Availability

The original contributions presented in the study are included in the article; further inquiries can be directed to the corresponding author.
